# The influence of perceived AI capability on learning engagement in smart education environments: the mediating role of academic self-efficacy and the moderating role of teacher-student relationship

**DOI:** 10.3389/fpsyg.2026.1790095

**Published:** 2026-05-22

**Authors:** Yufeng Xiang, Yongjun Zhang

**Affiliations:** 1 Faculty of humanities, The Education University of Hong Kong, Hong Kong, Hong Kong SAR, China; 2 International School, North China University of Technology, Beijing, China

**Keywords:** academic self-efficacy, generative artificial intelligence, higher education, learning engagement, mediation effect, moderated mediation effect, perceived AI capability, teacher-student relationship

## Abstract

**Objective:**

Based on social cognitive theory and conservation of resources theory, this study examines how perceived AI capability (PAC) relates to learning engagement (LE) in smart education environments. It focuses on the mediating role of academic self-efficacy (ASE) and the moderating role of teacher-student relationship (TSR).

**Methods:**

A three-wave longitudinal design was implemented over one academic semester. Participants were undergraduate students from a Beijing university (final *N* = 468) who consistently used an integrated intelligent learning system. PAC and TSR were measured at Time 1, ASE at Time 2, and LE at Time 3. Data were analyzed using structural equation modeling and bias-corrected bootstrapping (5,000 resamples).

**Results:**

PAC at Time 1 was positively associated with LE at Time 3 (*β* = 0.21, *p* < 0.001). ASE significantly mediated this relationship (indirect *β* = 0.19, 95% CI [0.13, 0.25]), accounting for 48% of the total effect. TSR moderated the PAC → ASE link (β_interaction = 0.12, *p* = 0.003). The conditional indirect effect was stronger under high TSR (b = 0.28, 95% CI [0.18, 0.38]) than under low TSR (b = 0.15, 95% CI [0.07, 0.23]), and the difference was significant (Δb = 0.13, 95% CI [0.04, 0.22]).

**Conclusion:**

Perceived AI capability is temporally associated with learning engagement, both directly and indirectly through academic self-efficacy. Positive teacher-student relationships strengthen this indirect pathway. Theoretically, this study moves beyond technology-centric views by treating PAC as a learner’s interpreted signal of a non-human agent’s competence—distinct from perceived usefulness or system quality—and by showing that AI’s psychological effects depend on human relational resources. Practically, institutions should support teacher-student rapport and self-efficacy alongside AI adoption. Intelligent systems need explainable, process-oriented feedback, and instructors should actively help students turn AI capability cues into lasting learning engagement.

## 1 Introduction

In recent years, generative AI and intelligent educational systems have rapidly entered higher education, reshaping how students acquire information, practice skills, and receive feedback (Haroud and Saqri, 2025). However, a key challenge has emerged. While student adoption of generative AI is increasing quickly (Tierney et al., 2025), low learning engagement (LE) remains a persistent problem. Research indicates that many students lack consistent engagement (Hrynowski, 2024), and engagement tends to decline across educational levels, with need-supportive teaching practices varying considerably (Patall et al., 2024). Thus, a central question arises: In smart education environments, through what psychological mechanisms is AI associated with LE, and under what conditions does this association vary?

Most existing studies focus on technology adoption or acceptance, not on LE itself—a construct more central to learning quality (Kemp et al., 2024). At the governance level, institutions are still adjusting to generative AI, with frameworks emphasizing human-centered goals (Holmes and Miao, 2023) and no stable consensus on AI’s role in learning (Burns and Muscanell, 2024). Some research suggests that answer-generating usage may undermine deep cognitive processing and independent thinking (Bastani et al., 2025; Appadoo-Ramsamy, 2025). These observations indicate that smart education research must move beyond descriptive questions of whether AI is used, toward mechanistic explanations of how perceived AI capability relates to learning beliefs and sustained LE (Heung and Chiu, 2025).

To address this gap, we introduce the concept of perceived AI capability (PAC). PAC refers to learners’ subjective evaluation of an intelligent system’s competency in learning support, including accuracy, adaptability, personalization, feedback timeliness, and stability (Marrone et al., 2025). Recent studies show that learners’ value judgments of generative AI are associated with motivation and usage strategies, while also revealing risks of over-reliance (Dogaru et al., 2025). Thus, AI likely operates as a perceived capability cue—an environmental signal that enters cognitive assessment processes (Berretta et al., 2023; Dorobăț et al., 2025). Pre−/post-semester studies show AI chat tools can affect LE, interest, and self-regulation, but effects depend heavily on context (Ng et al., 2024). Moreover, AI may boost short-term self-efficacy while undermining independent learning, threatening long-term sustainability (Zhai et al., 2024).

PAC is theoretically distinct from existing constructs such as perceived usefulness (task benefit), system quality (objective performance), or AI trust (reliability). Instead, PAC captures the learner’s *interpreted signal* of what the AI can do for them in a learning context. This distinction is critical for the present study.

From a theoretical perspective, social cognitive theory suggests that capability beliefs mediate the relationship between environmental cues and behavioral performance. Conservation of resources theory further emphasizes that external and internal resources underpin sustained investment (Hobfoll, 1989; Halbesleben et al., 2014; Alarcon et al.,2011). In smart education, academic self-efficacy (ASE) is a key psychological resource. Empirical research consistently shows a positive relationship between self-efficacy and LE, operating through motivation, flow, and emotion regulation (Zhou et al., 2025; Getenet et al., 2024). Applying these insights to AI contexts: when learners perceive an AI system as capable, they are more likely to internalize this external cue as a positive judgment of their own abilities, enhancing ASE and promoting subsequent LE. However, perceived benefits may coexist with risks of overdependence (Ogunleye et al., 2024).

Nevertheless, individual psychological mechanisms alone cannot fully account for heterogeneity in learning outcomes. A substantial body of research indicates that teacher-student relationship (TSR) is associated with how learners interpret feedback and persist through difficulties. In smart education, human-AI-teacher collaboration does not diminish the teacher’s role; rather, teachers’ social resources serve as important contextual conditions. Many students remain uncertain about acceptable AI use (Freeman, 2025). Empirical studies consistently show positive associations between TSR and LE (Tan et al., 2025), and structural models indicate that teacher support is positively associated with ASE and learning outcomes (Affuso et al., 2023). Therefore, TSR is a plausible boundary condition in the pathway from PAC to ASE and subsequently to LE.

Based on the above rationale, this study develops and tests a moderated mediation model. We hypothesize: (H1) PAC is positively associated with LE; (H2) ASE mediates this relationship; (H3) TSR moderates the PAC–ASE link, such that the positive association is stronger when TSR is high; and (H4) the indirect effect of PAC on LE via ASE is conditional on TSR, being stronger under high TSR. Using a three-wave longitudinal design and structural equation modeling, this study moves beyond a descriptive focus on technology use by examining how PAC relates to LE through psychological and relational pathways, and how TSR shapes the translation of AI capability cues into students’ efficacy beliefs and subsequent engagement.

## 2 Methodology

### 2.1 Research design

This study employed a three-wave longitudinal design to examine how PAC in smart education environments relates to LE, with ASE as a mediator and TSR as a moderator.

Three measurement points were spaced across one academic semester to align with the proposed temporal sequence. At Time 1 (T1, weeks 1–2), PAC and TSR were measured. At Time 2 (T2, weeks 5–6), ASE was measured. At Time 3 (T3, weeks 10–12), LE was measured. This temporal separation helps prevent conceptual overlap among key variables and supports the logic that contextual cues precede psychological resources, which in turn precede behavioral outcomes.

The design is grounded in social cognitive theory and conservation of resources theory. In smart education, PAC is conceptualized as an external contextual cue. According to these theories, such cues influence learners’ internal psychological resources (i.e., ASE), which subsequently shape sustained behavioral investment (i.e., LE). TSR is treated as a stable contextual resource that may moderate the PAC–ASE relationship. By measuring the independent variable, mediator, and dependent variable at different time points, the study reduces common method bias and provides stronger empirical support for the hypothesized temporal order compared to cross-sectional designs.

### 2.2 Participants and procedure

#### 2.2.1 Participants

Participants were undergraduate students from Northern China University of Technology who were using an intelligent educational system as part of their course studies. A cluster sampling method was used, with instructional classes serving as the sampling unit. Given the anonymous and low‑risk nature of the survey, written informed consent was not required. Participants’ voluntary completion and submission of the questionnaire constituted their implied informed consent, a procedure approved by the ethics committee and consistent with national research guidelines.

At Time 1 (T1), 620 questionnaires were distributed, with 587 valid responses returned (effective response rate: 94.7%). At Time 2 (T2), 512 valid responses were received, and at Time 3 (T3), 468 valid responses were obtained. Consequently, a total of 468 students who completed all three waves of measurement were included in the final longitudinal data analysis. The overall attrition rate was 20.3%, which falls within an acceptable range for longitudinal studies. In the final sample, the average student age was M = 19.8 years (SD = 1.1), with 46.6% male and 53.4% female participants. The sample included students from freshman to junior years, all of whom consistently used the course-associated intelligent educational system for learning activities throughout the study period.

**Figure 1:**
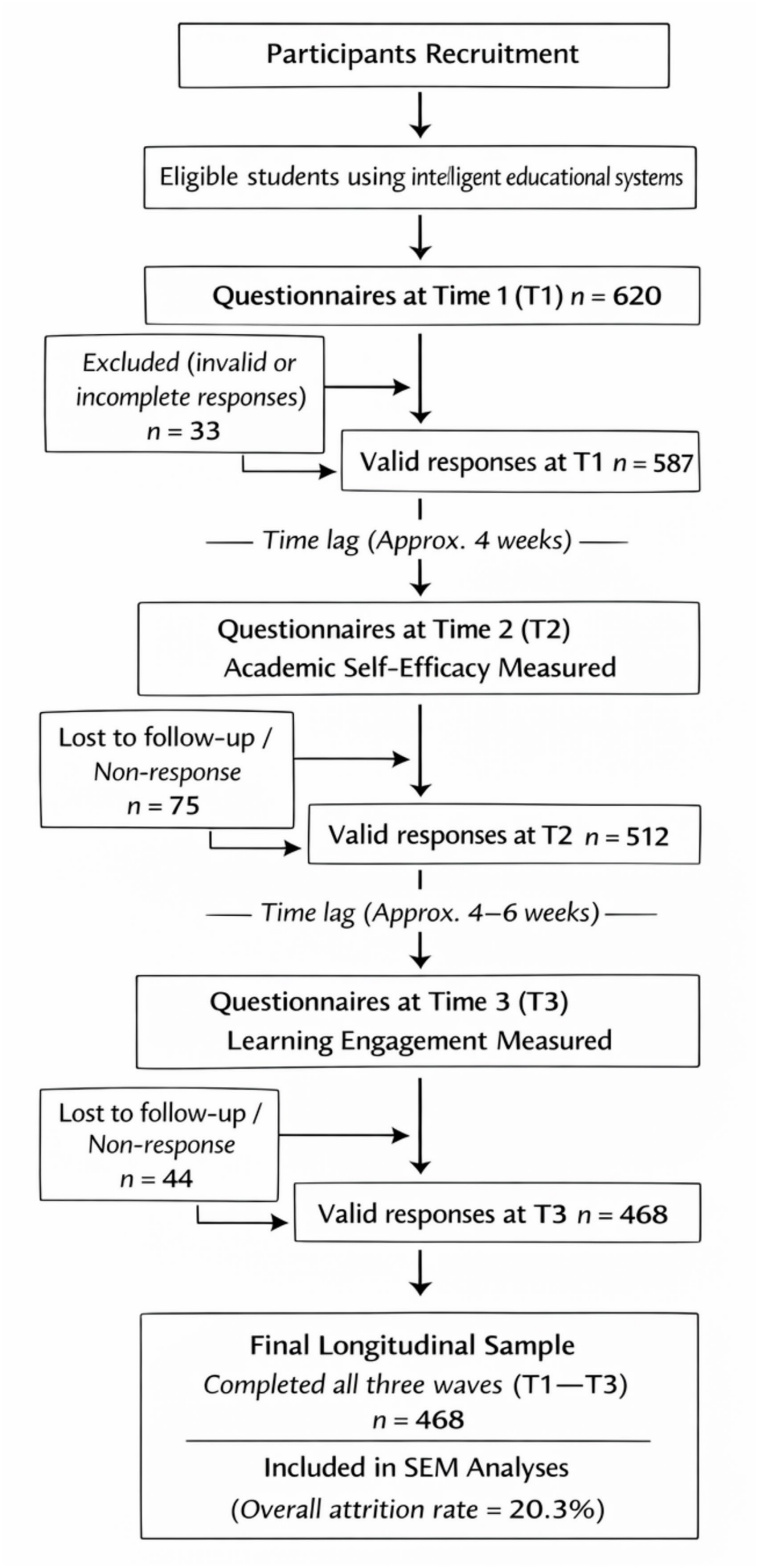
Flowchart of participant recruitment, follow-up, and final inclusion in the three-wave longitudinal study. This figure systematically presents the sample recruitment, data collection, and participant retention across the three measurement timepoints (T1–T3) within a single academic semester. At time 1 (T1), 620 questionnaires were distributed, yielding 587 valid responses. At time 2 (T2), 512 valid responses were received, and at time 3 (T3), 468 valid responses were collected. Ultimately, a total of 468 students completed all three waves of measurement and were included in the longitudinal structural equation modeling analysis, resulting in an overall participant attrition rate of 20.3%.

Given that sample attrition in longitudinal studies may introduce systematic bias, a comparative analysis was conducted prior to the main data analysis. This analysis compared participants who completed all three measurements with those who did not, on demographic variables (gender, grade) and key T1 variables (PAC, TSR). The results indicated no significant differences between the two groups on these variables (ps > 0.05), suggesting that attrition did not introduce significant systematic bias into the study findings. The study flowchart is shown in Figure 1.

#### 2.2.2 Procedure

A longitudinal questionnaire survey method was employed, with data collection conducted at three time points within a single semester to systematically examine the temporal sequence among core variables. Before data collection commenced, all participants were informed of the study’s purpose, procedures, data usage, and confidentiality principles. They were explicitly informed that participation was entirely voluntary and would not affect their course grades or academic evaluations.

To ensure accurate longitudinal data matching while preserving participant anonymity, a unique anonymous code was assigned to each participating student during the initial survey. This code, generated by the researchers solely for data linkage purposes, was used by students to complete questionnaires in subsequent waves. The researchers were unable to identify individual participants from these codes.

At Time 1 (T1, weeks 1–2 of the semester), the researchers administered the first questionnaire during relevant class sessions. The questionnaire primarily measured students’ perceptions of the AI capability within the course-associated intelligent educational system, their perceived TSR, and control variables. To reduce social desirability bias, the questionnaire cover page explicitly stated that there were no “right or wrong” answers and encouraged students to respond based on their genuine learning experiences. The first-wave questionnaire was typically completed in a classroom setting, taking approximately 10–15 min.

At Time 2 (T2, weeks 5–6 of the semester), the second survey was administered to the same cohort to measure ASE. This timing was chosen to ensure students had used the intelligent educational system for a sufficient period to form relatively stable perceptions of its features, allowing for an assessment of changes in their learning beliefs. The second questionnaire was distributed via an online platform, with students completing it within a specified timeframe (average completion time: 8–10 min).

At Time 3 (T3, weeks 10–12 of the semester), the third survey was conducted to measure students’ LE within the smart education environment. This later timing, when students were fully immersed in course activities, allowed for a more comprehensive self-assessment of their learning investment. The third questionnaire was also completed online, taking approximately 10–15 min.

To ensure contextual consistency and data quality for the longitudinal analysis, clear inclusion and exclusion criteria were established. Students included in the analysis were required to have continuously participated in the relevant course and used the associated intelligent educational system throughout the study period. Additionally, they needed to have completed the questionnaire at all three measurement points. Individuals who failed to complete any wave were excluded from the final longitudinal analysis. During data cleaning, questionnaire responses were screened for quality. Responses exhibiting clear patterns (e.g., straight-lining) or with substantial missing data on key variables were removed.

Through these procedures, the study achieved effective temporal separation in the measurement of core variables. This approach also minimized, at a procedural level, the potential impact of common method bias and invalid data on the results, thereby providing a reliable data foundation for the subsequent longitudinal model analysis.

### 2.3 Measures

All core variables were measured using structured self-report scales. Unless otherwise specified, items were rated on a five-point Likert scale ranging from 1 (strongly disagree) to 5 (strongly agree). Unless otherwise noted, scale scores were computed by averaging the corresponding items, with higher scores indicating higher levels of the construct.

To facilitate interpretation and descriptive analysis, and without implying any diagnostic or classificatory purpose, the mean scores for each scale were categorized into three ranges, following common practices in empirical research: 1.00–2.49 indicates a low level, 2.50–3.49 indicates a moderate level, and 3.50–5.00 indicates a high level. These ranges are used solely to aid in understanding sample characteristics, not for individual evaluation or inferential judgments.

All scales underwent a standardized localization process—“translation, back-translation, expert review, and pilot testing”—prior to the main survey. This ensured clarity of language, cultural appropriateness, and adequate content coverage. Unless otherwise noted, scores for each variable were calculated by averaging the corresponding items, with higher scores indicating a higher level of the construct.

#### 2.3.1 Perceived artificial intelligence capability (T1)

PAC measures learners’ subjective evaluation of the competency level demonstrated by an intelligent educational system in supporting their learning. This variable was measured at Time 1 (T1) to capture learners’ initial and relatively stable perceptions of system characteristics before the formation of subsequent learning beliefs.

##### 2.3.1.1 Scale composition and measurement content

Building upon prior research on AI capability perceptions and adapting it to the specific context, this study focused on system capabilities directly relevant to learning support. The scale comprises 10 items covering five dimensions: accuracy of system output, adaptability to learner differences, degree of personalization in learning support, timeliness of feedback and response, and reliability and stability of system operation. All items began with the contextual stem, “In the intelligent educational system used for this course…” to ensure judgments were based on a consistent learning context.

Examples of item content include: “The intelligent educational system can provide suitable learning suggestions based on my learning progress,” and “The learning feedback provided by the system is usually accurate and trustworthy.” These examples illustrate the nature of the items and do not constitute the complete scale.

To ensure that the PAC scale captures learners’ perceptions of AI capability rather than merely perceived usefulness or system quality, all items were framed with the behavioral learning context explicitly stated (e.g., “in the intelligent educational system used for this course”). Items focus on what the AI can do to support learning (e.g., providing adaptive suggestions, delivering accurate feedback), rather than whether the AI is useful, easy to use, or reliable. This contextualized and behavior-anchored approach helps maintain theoretical distinctiveness from general technology acceptance constructs.

To further clarify the theoretical positioning of PAC, Table 1 compares it with three established constructs commonly used in technology acceptance and human-AI interaction research.

**Table 1:** Conceptual distinctions between PAC and related constructs.

Construct	Definition focus	Theoretical role	Measurement emphasis
Perceived usefulness (TAM)	Usefulness for task performance	Attitude → Intention	Instrumental benefit
System quality (IS Success)	Objective or perceived system performance	Information/Service quality	Accuracy, reliability, ease of use
AI trust (HCI)	Reliability, predictability, integrity	Risk reduction, acceptance	Confidence in AI’s actions
Perceived AI capability (PAC)	AI’s competence in learning support	Capability cue → Self-efficacy → Engagement	Personalization, feedback accuracy, adaptability, responsiveness

##### 2.3.1.2 Scoring standards and rules

Each item was rated on the five-point Likert scale as described. The overall PAC score is the mean of all 10 items, with higher scores indicating a higher perceived level of AI capability. For dimensional-level analyses, sub-dimension scores are the means of their respective items.

##### 2.3.1.3 Interpretation and measurement notes

In descriptive statistics, a PAC mean in the 1.00–2.49 range is interpreted as a low perception level, 2.50–3.49 as moderate, and 3.50–5.00 as high. This interpretation serves only to summarize the overall perceptual characteristics of the sample and does not constitute an objective evaluation of system performance. The reliability and validity of the scale are reported in the subsequent analysis section.

#### 2.3.2 Academic self-efficacy (T2)

ASE reflects students’ beliefs in their ability to complete learning tasks, cope with learning challenges, and sustain engagement in learning activities. This variable was measured at Time 2 (T2), consistent with the theoretical sequence of “contextual cues influencing individual beliefs.”

##### 2.3.2.1 Scale composition and measurement content

An established ASE scale was adapted to the smart education context. The scale consists of 6 items, primarily covering students’ beliefs regarding understanding course content, completing learning tasks, persisting when encountering difficulties, and improving learning performance through effort. Item design emphasized capability beliefs themselves, avoiding direct overlap with outcome behaviors like learning engagement.

Example items include: “Even when the learning material is complex, I am confident I can understand and master it through effort,” and “When I encounter difficulties during learning, I believe I can find solutions.”

##### 2.3.2.2 Scoring standards and rules

All items used the five-point Likert scale. The ASE score is the mean of the 6 items, with higher scores indicating higher ASE. If any reverse-coded items were present, they were recorded during data preparation to ensure uniform scoring direction.

##### 2.3.2.3 Interpretation and measurement notes

The interpretation of ASE mean ranges at the sample level follows the same low-moderate-high standard described earlier, used solely to present the general level of self-efficacy in the sample, not to judge individual learning ability. The scale’s structural soundness and internal consistency are verified in subsequent analyses.

#### 2.3.3 Learning engagement (T3)

LE characterizes the degree of student investment in learning within the smart education environment and is a primary outcome variable in this study. Measured at Time 3 (T3), it reflects students’ comprehensive engagement state after experiencing a period of learning.

##### 2.3.3.1 Scale composition and measurement content

Drawing on mainstream LE theory, this study measured engagement across three dimensions: behavioral, emotional, and cognitive. The scale contains 12 items, with 4 items per dimension. Behavioral engagement pertains to the continuity and completion of learning behaviors; emotional engagement pertains to interest and positive emotional experiences during learning; cognitive engagement pertains to deep processing, reflection, and the use of learning strategies.

Example items include: “When learning with this intelligent educational system, I proactively invest time to complete relevant learning tasks,” and “During the learning process, I think about how to connect new knowledge with prior knowledge.”

##### 2.3.3.2 Scoring standards and rules

All items used the five-point Likert scale. Dimension scores are the means of their respective items, and the overall LE score is the mean of all 12 items. Higher scores indicate greater learning engagement. In structural model analysis, LE can be modeled as a higher-order latent variable, as detailed later.

##### 2.3.3.3 Interpretation and measurement notes

The interpretation of LE score ranges similarly follows the descriptive low-moderate-high classification, used only to depict overall sample characteristics. Scale design distinguished between “learning investment behaviors” and “capability beliefs” to minimize potential conceptual overlap with ASE.

#### 2.3.4 Teacher-student relationship (T1)

TSR reflects students’ overall perception of teacher support, understanding, and interaction quality. It is a key contextual variable in this study, measured at Time 1 (T1) and serving as a moderator in the theoretical model.

##### 2.3.4.1 Scale composition and measurement content

Adapted from established scales on teacher support and relationship quality, this scale comprises 8 items. It primarily covers teacher care and respect, the availability of supportive feedback, and trust and communication quality within teacher-student interactions. Items avoided evaluating teaching effectiveness to maintain focus on the relational and supportive resource aspects of the construct.

Example items include: “During the learning process, the teacher is usually willing to provide support when I need help,” and “I can feel the teacher’s respect and understanding for students in class or during learning activities.”

##### 2.3.4.2 Scoring standards and rules

Items used the five-point Likert scale. The TSR score is the mean of the 8 items, with higher scores indicating a higher perceived quality of the TSR. Any reverse-coded items were uniformly recoded prior to scoring.

##### 2.3.4.3 Interpretation and measurement notes

In descriptive analysis, the interpretation of TSR score ranges follows the same low-moderate-high classification, used solely to present the sample’s general perception of this relationship. As a relatively stable contextual resource variable, measuring TSR at T1 helps establish a clear temporal distinction from subsequent psychological and behavioral variables.

### 2.4 Control variables

To reduce the influence of potential confounding factors on the study’s conclusions and enhance the internal validity of model estimates, a set of demographic and learning background variables potentially related to LE and its antecedents were included as controls in the structural model analysis. Control variable selection followed principles of clear theoretical relevance, occurrence prior to core variables, and demonstrated potential influence in prior research, to avoid over-control or the introduction of irrelevant variables.

Specifically, the study controlled for gender, grade level, prior academic achievement, and prior experience with intelligent learning systems. These variables were all measured at Time 1 (T1) to ensure they preceded the core independent, mediator, and dependent variables in time, aligning with the basic requirement for control variable temporality in longitudinal research.

Operationally, gender was measured as a categorical variable and coded using a dummy variable (Gender was coded as 0 = male, 1 = female.) in the analysis. Grade level, serving as an indicator of academic stage and development, was included as an ordinal variable, with higher values indicating higher grade levels. Prior academic achievement, reflecting students’ learning foundation and existing academic ability, was measured via student self-report of recent overall academic performance—a measure widely used in educational research to control for individual academic level differences. Prior experience with intelligent learning systems gauged students’ familiarity with relevant technology before the study began, typically measured via self-report of past frequency or duration of use of such systems.

During model estimation, these control variables were simultaneously included in the structural model and specified as exogenous variables with direct effects on ASE and learning engagement. This was done to account for their potential confounding influence on the estimation of core pathways. By systematically controlling for these factors, the study aimed to more accurately examine the theoretical relationships among PAC, ASE, and learning engagement, thereby strengthening the robustness and explanatory power of the findings.

### 2.5 Data analysis

Structural Equation Modeling (SEM) was employed to test the research hypotheses. SEM is capable of simultaneously handling latent variable measurement error and structural path relationships, making it particularly suitable for analyzing complex theoretical models involving mediation and moderation effects. All model estimations were performed using Mplus 8.0. Parameter estimation methods, including Maximum Likelihood (ML) or its robust variants, were selected based on data characteristics.

#### 2.5.1 Measurement model assessment

Prior to structural model analysis, Confirmatory Factor Analysis (CFA) was conducted for all latent variables to assess the reliability and validity of the measurement model. Specifically, the internal consistency reliability, convergent validity, and discriminant validity of each latent variable were evaluated to ensure the statistical distinctiveness of the different constructs.

At the overall measurement model level, goodness-of-fit indices were used to assess model-data fit. Commonly used indices included the chi-square statistic (χ^2^), Comparative Fit Index (CFI), Tucker-Lewis Index (TLI), Root Mean Square Error of Approximation (RMSEA), and Standardized Root Mean Square Residual (SRMR). The interpretation of these indices followed standard conventions in SEM. Furthermore, the fit of the theoretical measurement model was compared with that of alternative models (e.g., factor combination models or a single-factor model) to further verify its structural soundness and provide a reliable basis for subsequent path analysis.

#### 2.5.2 Longitudinal structural model and mediation effect testing

Upon establishing support for the measurement model, a longitudinal structural model was constructed to test the research hypotheses. According to the research design, PAC (T1) was specified as the independent variable, ASE (T2) as the mediator, and LE (T3) as the dependent variable, establishing a clear temporal order and causal direction among variables.

First, the direct effect of PAC on LE was tested. Then, ASE was introduced into the model to test its mediating role in the relationship between PAC and learning engagement. The significance of the mediation effect was assessed using the bias-corrected bootstrap method, with 5,000 resamples. An indirect effect was considered statistically significant if its 95% confidence interval did not include zero. This method is considered robust for testing mediation as it does not rely on the normality assumption.

#### 2.5.3 Moderation and moderated mediation model testing

To test the moderating effect of TSR on the relationship between PAC and ASE, an interaction term between PAC and TSR was introduced into the structural model, with ASE as the dependent variable. To reduce multicollinearity risk, the relevant variables were mean-centered before creating the interaction term.

Subsequently, moderated mediation was tested—that is, whether the TSR influences the strength of the indirect path from PAC to LE via ASE. Conditional indirect effects were also estimated using the bias-corrected bootstrap method and compared across different levels of TSR (e.g., mean ±1 standard deviation). A moderated mediation effect is supported if the indirect effects are significant and differ across conditions.

#### 2.5.4 Control variables and model specification

In all structural models, gender, grade level, prior academic achievement, and prior experience with intelligent learning systems were included simultaneously as control variables, specified as exogenous variables with direct effects on ASE and learning engagement. This approach statistically accounted for the potential confounding influence of these factors on the core path relationships, thereby improving the internal validity of the parameter estimates.

#### 2.5.5 Missing data handling and estimation robustness

To address the inevitable missing data in longitudinal studies, Full Information Maximum Likelihood (FIML) estimation was employed. Under the assumption that data are Missing at Random (MAR), FIML utilizes all available information for parameter estimation and is considered a recommended method for handling missing data in longitudinal SEM. Additionally, prior to final reporting, checks were performed on the reasonableness and stability of the model estimates. This included checking for abnormal parameter estimates, the reasonableness of standard errors, and any obvious model identification issues. These steps helped ensure the robustness and replicability of the study conclusions.

### 2.6 Common method bias

Given that some variables were collected via self-report questionnaires, there was a potential risk of Common Method Bias (CMB). Accordingly, strategies to control for CMB were incorporated at the research design stage, and supplementary statistical checks were conducted during data analysis.

At the research design level, the three-wave longitudinal design temporally separated the measurement of core variables—measuring the independent, mediator, and dependent variables at different time points. This procedural separation helps weaken the risk of systematic covariation arising from identical measurement contexts and response sources. Temporal separation is widely recognized as an effective strategy for mitigating CMB, particularly in longitudinal studies involving psychological mechanism variables.

At the statistical testing level, Confirmatory Factor Analysis was further used to assess the potential for CMB. Specifically, a single-factor model, where all measurement items loaded onto a single latent factor, was compared with the theoretical multi-factor measurement model in terms of goodness-of-fit. The results showed that the single-factor model fit was significantly worse than the multi-factor model, while the theoretical model exhibited good fit.

Considering the outcomes of both the procedural controls and statistical tests, common method bias does not appear to pose a major threat to the validity of the findings within the context of this study. Its potential influence on the estimation of relationships among core variables is likely limited, suggesting that the measurement foundation for the subsequent longitudinal structural model analysis is reasonably acceptable.

## 3 Results

### 3.1 Preliminary analyses

Prior to formal hypothesis testing, preliminary statistical analyses were conducted on the study data. Descriptive statistics, including means (M), standard deviations (SD), and Pearson correlation coefficients among key study variables, were computed. The results are presented in Table 2.

**Table 2:** Descriptive statistics, reliabilities, and cross-time correlations among study variables.

Variable	M	SD	1	2	3	4	5	6	7	8
1. Perceived artificial intelligence capability (T1)	3.62	0.71	(0.89)							
2. Academic self-efficacy (T2)	3.58	0.68	0.46***	(0.87)						
3. Learning engagement (T3)	3.65	0.64	0.39***	0.53***	(0.91)					
4. Teacher-student relationship (T1)	3.74	0.66	0.34***	0.41***	0.44***	(0.88)				
5. Gender (T1)	0.53	0.50	0.05	0.07	0.06	0.04	—			
6. Grade (T1)	2.14	0.76	0.09	0.10	0.12*	0.08	0.03	—		
7. Prior academic achievement (T1)	3.41	0.72	0.18**	0.21**	0.18**	0.16**	0.02	0.19**	—	
8. Prior AI system experience (T1)	2.87	0.81	0.24***	0.15**	0.13*	0.17**	0.01	0.11*	0.20**	—

*N* = 468. Values on the diagonal in parentheses are Cronbach’s α coefficients. Gender was coded as 0 = male, 1 = female. Grade represents students’ year of study. Prior academic achievement and prior AI system experience were self-reported at Time 1. Correlations among core study variables represent cross-time associations (T1-T2-T3). All correlation coefficients are below 0.60, indicating no serious multicollinearity concerns. T1 = Time 1; T2 = Time 2; T3 = Time 3. *p < 0.05, **p < 0.01, ***p < 0.001.

The findings indicate that learners’ PAC (T1) was at a moderately high level (M = 3.62, SD = 0.71). ASE (T2) averaged 3.58 (SD = 0.68), LE (T3) averaged 3.65 (SD = 0.64), and TSR (T1) averaged 3.74 (SD = 0.66). These results suggest that the sample generally held favorable evaluations of the smart education environment and demonstrated moderate to high levels of learning beliefs and investment.

**Figure 2:**
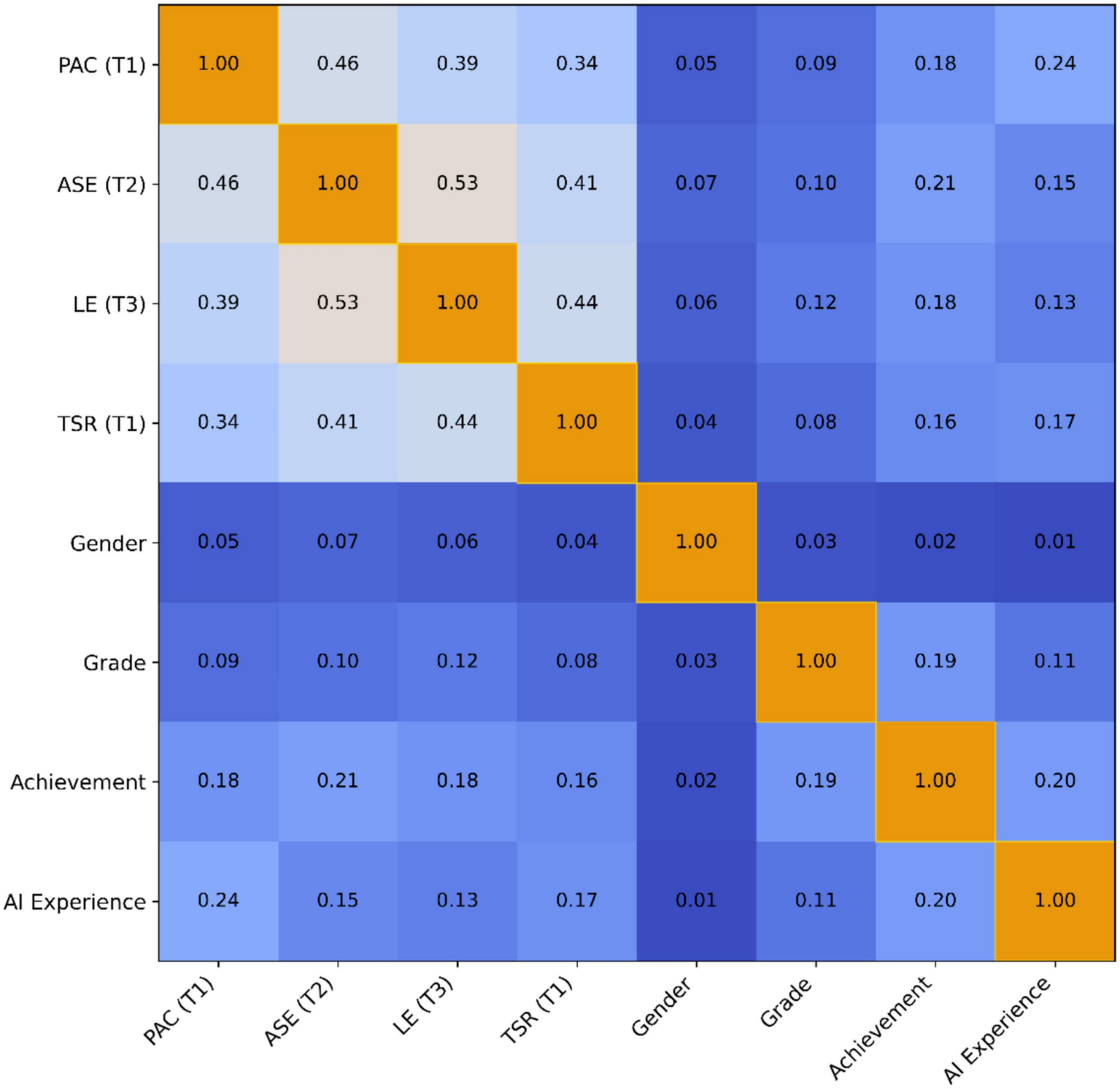
Correlation matrix of the primary study variables. Pearson correlation coefficients among perceived artificial intelligence capability (T1), academic self-efficacy (T2), learning engagement (T3), and teacher-student relationship (T1). The color intensity reflects the magnitude and direction of the correlation coefficients. The diagonal displays the internal consistency reliability (Cronbach’s *α*) for each variable. This figure illustrates the overall correlational structure among the key study variables.

Correlation analyses revealed a significant positive correlation between PAC and ASE (r = 0.46, *p* < 0.001), as well as a significant positive correlation with LE (r = 0.39, *p* < 0.001). The correlation between ASE and LE was r = 0.53 (*p* < 0.001), indicating a strong positive association. Furthermore, TSR showed significant positive correlations with PAC (r = 0.34, *p* < 0.001), ASE (r = 0.41, *p* < 0.001), and LE (r = 0.44, *p* < 0.001) (see Figure 2). The directions of these correlations are consistent with the research hypotheses, providing initial empirical support for the subsequent structural model analysis.

Further examination of the distributional characteristics of the main study variables showed that the absolute values of skewness for all variables were below 1.00, and the absolute values of kurtosis were below 1.20. These values fall within the generally acceptable range for structural equation modeling (SEM) analyses, indicating that the data did not substantially deviate from the assumption of normality and were suitable for parameter estimation using maximum likelihood methods. Correlations between control variables and the main study variables were also examined. Results showed small correlations between gender and the main variables (|r| ≤ 0.08, ps > 0.05). Grade level showed a weak positive correlation with LE (r = 0.12, *p* < 0.05). Prior academic achievement showed significant, albeit moderate-weak, correlations with ASE (r = 0.21, *p* < 0.01) and LE (r = 0.18, *p* < 0.01). Additionally, prior experience with intelligent learning systems showed a significant positive correlation with PAC (r = 0.24, *p* < 0.001), but its correlations with other main study variables did not exceed 0.30. Overall, the correlations between control variables and core variables were within reasonable limits, showing no serious risk of multicollinearity.

In summary, the preliminary analysis results indicate that the study data meet the basic assumptions for subsequent SEM analysis in terms of both distributional characteristics and variable relationships.

### 3.2 Measurement model

**Figure 3:**
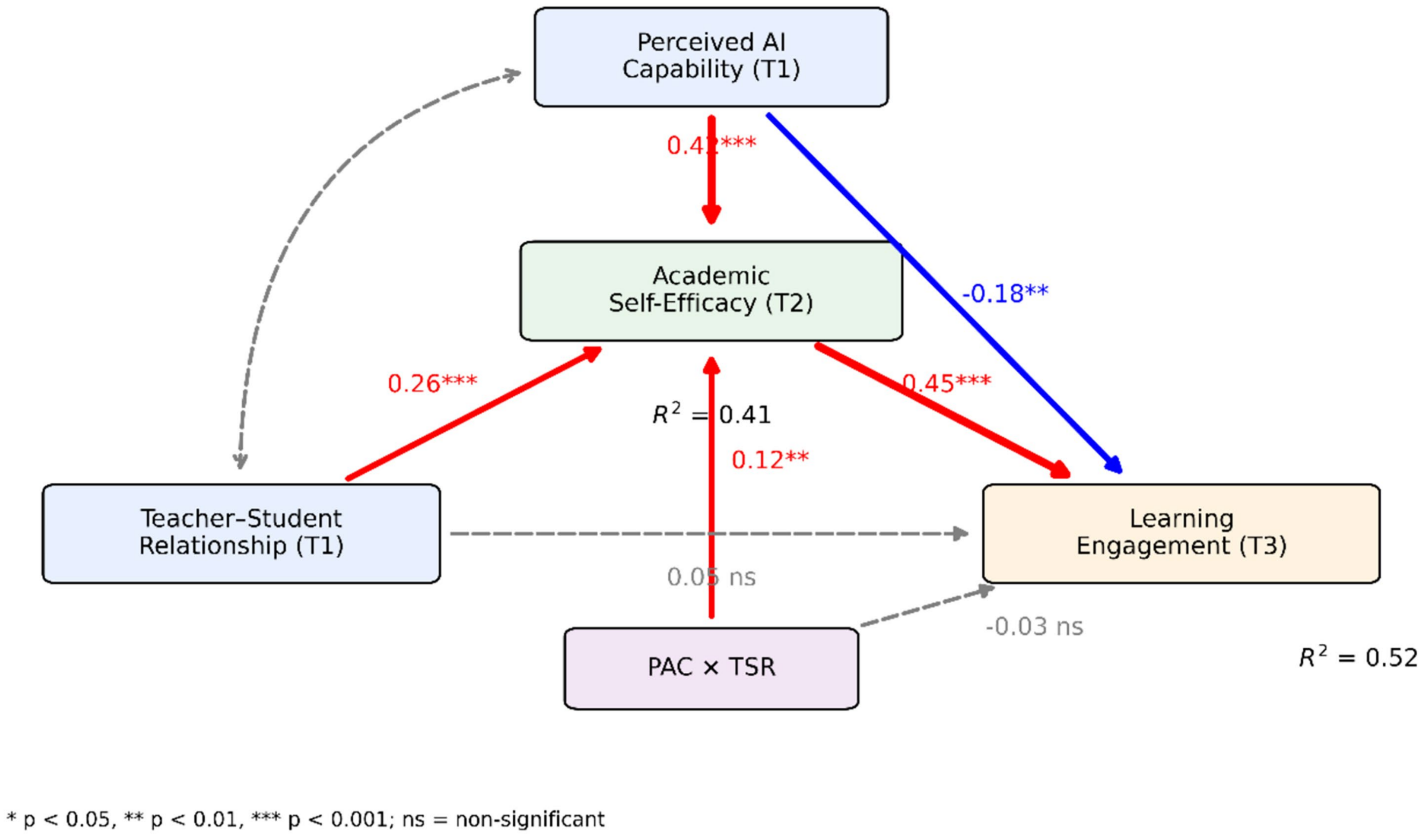
Longitudinal structural model of perceived AI capability, academic self-efficacy, and learning engagement. The longitudinal structural model constructed in this study, depicting the structural relationships among perceived AI capability (T1), academic self-efficacy (T2), learning engagement (T3), and teacher-student relationship (T1). Path coefficients shown are standardized estimates. Solid lines represent statistically significant paths; dashed lines represent non-significant paths. The model controls for gender, grade level, prior academic achievement, and prior experience with intelligent learning systems.

To assess the reliability and validity of the measurement instruments, a Confirmatory Factor Analysis (CFA) was first conducted on the measurement model encompassing PAC, ASE, LE (with its dimensions: Behavioral, Emotional, and Cognitive Engagement), and TSR. LE was modeled as a second-order latent variable reflected by the three first-order latent variables (Behavioral, Emotional, and Cognitive Engagement), reflecting its multidimensional structure (see Figure 3).

The CFA results indicated that the theoretical measurement model met commonly used criteria for good fit in SEM research. Specifically, the model fit statistics were: χ^2^ = 612.47, df = 344, χ^2^/df = 1.78, Comparative Fit Index (CFI) = 0.95, Tucker-Lewis Index (TLI) = 0.94, Root Mean Square Error of Approximation (RMSEA) = 0.041 (90% CI = [0.036, 0.046]), and Standardized Root Mean Square Residual (SRMR) = 0.045. These fit indices suggest that the measurement model adequately reproduced the sample covariance matrix, indicating an overall good fit (see Table 3).

**Table 3:** Measurement model fit and construct validity.

Construct/model	χ^2^	df	χ^2^/df	CFI	TLI	RMSEA	SRMR	Std. loadings	CR	AVE
Measurement models										
Theoretical measurement model	612.47	344	1.78	0.95	0.94	0.041	0.045	0.68–0.86 (min = 0.68)	—	—
One-factor model	1486.32	350	4.25	0.61	0.58	0.098	0.112	—	—	—
PAC and TSR combined model	801.36	350	2.29	0.86	0.84	0.062	0.071	—	—	—
Constructs										
Perceived artificial intelligence capability (T1)	—	—	—	—	—	—	—	0.71–0.84 (min = 0.71)	0.89	0.61
Academic self-efficacy (T2)	—	—	—	—	—	—	—	0.68–0.82 (min = 0.68)	0.87	0.56
Behavioral engagement (T3)	—	—	—	—	—	—	—	0.72–0.86 (min = 0.72)	0.91	0.68
Emotional engagement (T3)	—	—	—	—	—	—	—	0.70–0.83 (min = 0.70)	0.88	0.59
Cognitive engagement (T3)	—	—	—	—	—	—	—	0.69–0.85 (min = 0.69)	0.93	0.69
Teacher-student relationship (T1)	—	—	—	—	—	—	—	0.73–0.86 (min = 0.73)	0.90	0.64
Learning engagement (second-order)	—	—	—	—	—	—	—	—	0.92	0.66

PAC refers to perceived artificial intelligence capability, and TSR refers to teacher-student relationship. All standardized factor loadings are statistically significant at *p* < 0.001. CR denotes composite reliability, and AVE denotes average variance extracted. Learning engagement is specified as a second-order construct reflected by behavioral, emotional, and cognitive engagement.

At the item level, all standardized factor loadings on their respective latent variables were statistically significant (ps < 0.001), ranging from 0.68 to 0.86, exceeding the commonly adopted minimum threshold in SEM analyses. Further calculations showed Composite Reliability (CR) values ranging from 0.87 to 0.93 and Average Variance Extracted (AVE) values ranging from 0.56 to 0.69 (see Table 3). These indices suggest that the latent variables exhibited acceptable levels of internal consistency and convergent validity.

To test for discriminant validity, the theoretical measurement model was compared with several alternative models. Specifically, models were constructed by merging PAC with TSR, merging ASE with Learning Engagement, and loading all measurement items onto a single latent factor (single-factor model). Model comparison results showed that the fit indices for these alternative models were significantly worse than those for the theoretical measurement model, with chi-square difference tests reaching statistical significance (Δχ^2^, ps < 0.001; see Table 3), thus supporting the statistical distinctiveness of the study constructs.

In summary, the CFA results demonstrate that the measurement model in this study met acceptable standards for reliability, convergent validity, and discriminant validity in SEM analysis, providing a robust measurement foundation for subsequent structural model analysis and hypothesis testing.

### 3.3 Structural model and direct effects

A longitudinal structural model was constructed to test the research hypotheses, with PAC (T1) as the independent variable, ASE (T2) as the mediator, and LE (T3) as the dependent variable. Control variables (gender, grade level, prior academic achievement, and prior AI system experience) were included. Parameters were estimated using maximum likelihood.

The structural model demonstrated good fit: χ^2^ = 684.92, df = 362, χ^2^/df = 1.89, CFI = 0.94, TLI = 0.93, RMSEA = 0.043 (90% CI [0.038, 0.048]), SRMR = 0.047 (Figure 3). A reverse path from ASE (T2) to PAC (T1) was also estimated. This path was statistically significant (*β* = −0.18, SE = 0.06, *p* < 0.01), suggesting a potential bidirectional relationship between perceived AI capability and academic self-efficacy. Given this finding, the specified temporal direction should be interpreted with caution, and causal claims are not warranted based solely on the current design.

As shown in Table 4, PAC showed a significant positive direct association with LE (*β* = 0.21, SE = 0.05, *p* < 0.001) and on ASE (*β* = 0.42, SE = 0.04, *p* < 0.001). TSR also showed a significant positive direct effect on ASE (*β* = 0.26, SE = 0.04, *p* < 0.001) (Figure 3).

**Table 4:** Structural model path estimates for direct effects.

Outcome	Predictor	b	SE(b)	95% CI for b	β	SE(β)	z	*p*
Learning Engagement (T3)	Perceived AI capability (T1)	0.24	0.06	[0.12, 0.36]	0.21	0.05	4.20	< 0.001
	Academic self-efficacy (T2)	0.48	0.05	[0.38, 0.58]	0.45	0.05	9.00	< 0.001
	Grade level (T1)	0.09	0.04	[0.01, 0.17]	0.11	0.05	2.20	0.028
	Prior academic achievement (T1)	0.14	0.04	[0.06, 0.22]	0.16	0.05	3.20	0.001
	Gender (T1)	−0.03	0.04	[−0.11, 0.05]	−0.03	0.04	−0.75	0.453
	AI system experience (T1)	0.05	0.05	[−0.05, 0.15]	0.05	0.05	1.00	0.317
Academic Self-Efficacy (T2)	Perceived AI Capability (T1)	0.46	0.04	[0.38, 0.54]	0.42	0.04	10.50	< 0.001
	Prior academic achievement (T1)	0.17	0.05	[0.07, 0.27]	0.19	0.06	3.17	0.002
	Gender (T1)	0.04	0.04	[−0.04, 0.12]	0.04	0.04	1.00	0.317
	AI system experience (T1)	0.06	0.05	[−0.04, 0.16]	0.06	0.05	1.20	0.230
Perceived AI Capability (T1)	AI system experience (T1)	0.31	0.07	[0.17, 0.45]	0.23	0.05	4.60	< 0.001

b = unstandardized path coefficient. β = standardized path coefficient. SE = standard error. Confidence intervals are 95% normal-theory intervals. Gender was coded as 0 = male, 1 = female. All control variables were measured at T1 and included as exogenous predictors. Non-significant effects are retained.

Regarding control variables, grade level showed a weak positive association with LE (*β* = 0.11, *p* < 0.05). Prior academic achievement was positively associated with both ASE (*β* = 0.19, *p* < 0.01) and LE (*β* = 0.16, *p* < 0.01). Prior AI system experience was positively associated with PAC (*β* = 0.23, *p* < 0.001). Gender effects were not significant. Inclusion of control variables did not substantially alter the core structural relationships.

In summary, the longitudinal structural model shows that PAC is positively associated with subsequent LE and ASE, consistent with a temporal precedence interpretation, though causal direction cannot be firmly established. The significant reverse path from ASE (T2) to PAC (T1) further suggests bidirectionality, reinforcing that these findings are associative rather than causal.

### 3.4 Mediation analysis

Following the support for direct effects in the structural model, the mediating role of ASE in the relationship between PAC and LE was further examined. The mediation effect was estimated using the bias-corrected bootstrap method, based on 5,000 resamples, with 95% confidence intervals as the criterion for significance.

As shown in Table 5, both the a-path (PAC → ASE) and b-path (ASE → LE) were statistically significant (*β* = 0.42 and 0.45, respectively, both *p* < 0.001).

**Table 5:** Comprehensive mediation analysis of academic self-efficacy.

Component	Effect	b	SE/Boot SE	95% CI for b	β	95% CI for β	z	*p*
Path estimates	a path PAC (T1) → ASE (T2)	0.46	0.04	[0.38, 0.54]	0.42	[0.34, 0.50]	10.50	< 0.001
	b path ASE (T2) → LE (T3)	0.48	0.05	[0.38, 0.58]	0.45	[0.35, 0.55]	9.00	< 0.001
	c′ path PAC (T1) → LE (T3)	0.24	0.06	[0.12, 0.36]	0.21	[0.11, 0.31]	4.20	< 0.001
Effect decomposition	Indirect effect a × b	0.22	0.04	[0.14, 0.30]	0.19	[0.13, 0.25]	6.33	< 0.001
	Direct effect c′	0.24	0.06	[0.12, 0.36]	0.21	[0.11, 0.31]	4.20	< 0.001
	Total effect c	0.46	0.06	[0.34, 0.58]	0.40	[0.32, 0.48]	10.00	< 0.001
Relative contribution	Proportion mediated (VAF)	0.48	0.07	[0.33, 0.62]	0.48	[0.33, 0.62]	6.86	< 0.001
	Indirect/direct effect ratio	0.90	0.18	[0.62, 1.28]	—	—	5.00	< 0.001

PAC denotes perceived AI capability, ASE denotes academic self-efficacy, and LE denotes learning engagement. b represents unstandardized effects and β represents standardized effects. Standard errors for indirect and total effects are bootstrap-based. Confidence intervals for indirect and total effects are bias-corrected (BC) 95% bootstrap intervals based on 5,000 resamples. Gender, grade level, prior academic achievement, and prior AI system experience were included as control variables in all equations but are omitted from the table for clarity. VAF = variance accounted for, representing the proportion of the total effect mediated.

Based on this, bootstrap testing for the indirect effect was conducted. The results showed that the indirect effect of PAC on LE through ASE was statistically significant, with an unstandardized estimate of b = 0.22, Boot SE = 0.04, bias-corrected 95% CI = [0.14, 0.30]. The corresponding standardized indirect effect was *β* = 0.19, 95% CI = [0.13, 0.25]. Since these confidence intervals did not include zero, it can be concluded that ASE played a significant mediating role between PAC and LE (see Table 5).

After including ASE as a mediator, the direct effect of PAC on LE remained significant (unstandardized path coefficient b = 0.24, SE = 0.06, 95% CI = [0.12, 0.36], standardized coefficient *β* = 0.21, *p* < 0.001). Further decomposition of effects showed that the total effect of PAC on LE was b = 0.46, 95% CI = [0.34, 0.58], of which approximately 48% was mediated indirectly through ASE (variance accounted for, VAF = 0.48, 95% CI = [0.33, 0.62]). This indicates that ASE functioned as a partial mediator in the relationship (see Table 5).

**Figure 4:**
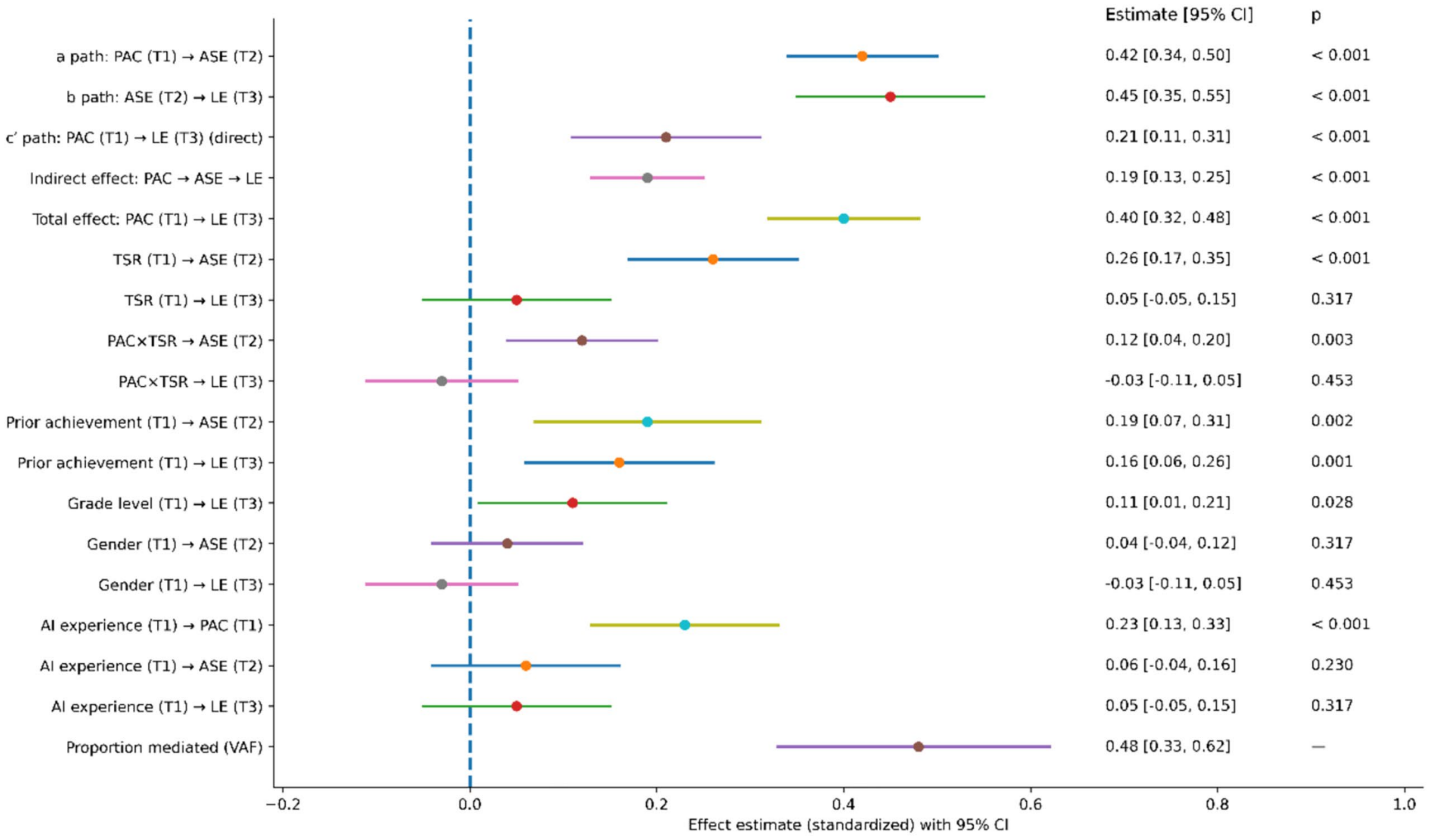
Direct, indirect, and total effects of perceived AI capability on learning engagement. Point estimates and corresponding 95% confidence intervals for the direct effect of perceived AI capability on learning engagement, its indirect effect via academic self-efficacy, and the total effect. The vertical reference line indicates the null effect level. This figure aids in assessing the direction, magnitude, and statistical robustness of the mediation effect.

To assess the robustness of the mediation effect, the key path coefficients, direct effect, indirect effect, and their confidence intervals are presented comprehensively (see Figure 4). The results show that the indirect effect remained positive and significant across different statistical indicators, consistent with the numerical results in Table 5, further supporting the mediating role of ASE.

### 3.5 Moderation and moderated mediation effects

To examine the moderating role of TSR on the relationship between PAC and ASE, an interaction term (PAC × TSR) was introduced into the structural model with ASE as the dependent variable. (Table 6).

**Table 6:** Moderation and moderated mediation effects of teacher-student relationship.

Component	Effect	b	SE/Boot SE	95% CI for b	*β*	95% CI for β	z	*p*
Moderation (interaction term)	PAC (T1) × TSR (T1) → ASE (T2)	0.15	0.05	[0.05, 0.25]	0.12	[0.04, 0.20]	3.00	0.003
Simple slopes	PAC (T1) → ASE (T2) at TSR High (+1 SD)	0.58	0.06	[0.46, 0.70]	0.53	[0.42, 0.64]	9.67	< 0.001
	PAC (T1) → ASE (T2) at TSR Low (−1 SD)	0.31	0.07	[0.17, 0.45]	0.28	[0.15, 0.41]	4.43	< 0.001
Conditional indirect effects (BC bootstrap)	Indirect effect PAC → ASE → LE at TSR High (+1 SD)	0.28	0.05	[0.18, 0.38]	0.24	[0.16, 0.32]	—	—
	Indirect effect PAC → ASE → LE at TSR Low (−1 SD)	0.15	0.04	[0.07, 0.23]	0.13	[0.06, 0.20]	—	—
Moderated mediation test	Difference in conditional indirect effects (High − Low)	0.13	0.05	[0.04, 0.22]	0.11	[0.04, 0.18]	—	—
	Index of moderated mediation	0.07	0.03	[0.02, 0.13]	0.06	[0.02, 0.11]	—	—

PAC denotes perceived AI capability, TSR denotes teacher-student relationship, ASE denotes academic self-efficacy, and LE denotes learning engagement. b represents unstandardized effects and β represents standardized effects. Conditional indirect effects and the index of moderated mediation were evaluated using bias-corrected (BC) bootstrap confidence intervals based on 5,000 resamples. For bootstrap-based quantities, z and p values are not reported.

The interaction term was significantly associated with ASE (b = 0.15, SE = 0.05, 95% CI [0.05, 0.25], *β* = 0.12, *p* = 0.003), suggesting that TSR moderated the PAC–ASE relationship. Simple slope analyses revealed that under high TSR conditions (mean +1 SD), the positive association between PAC and ASE was significantly stronger (b = 0.58, SE = 0.06, *p* < 0.001); under low TSR conditions (mean −1 SD), the association remained positive but notably weaker (b = 0.31, SE = 0.07, *p* < 0.001).

**Figure 5:**
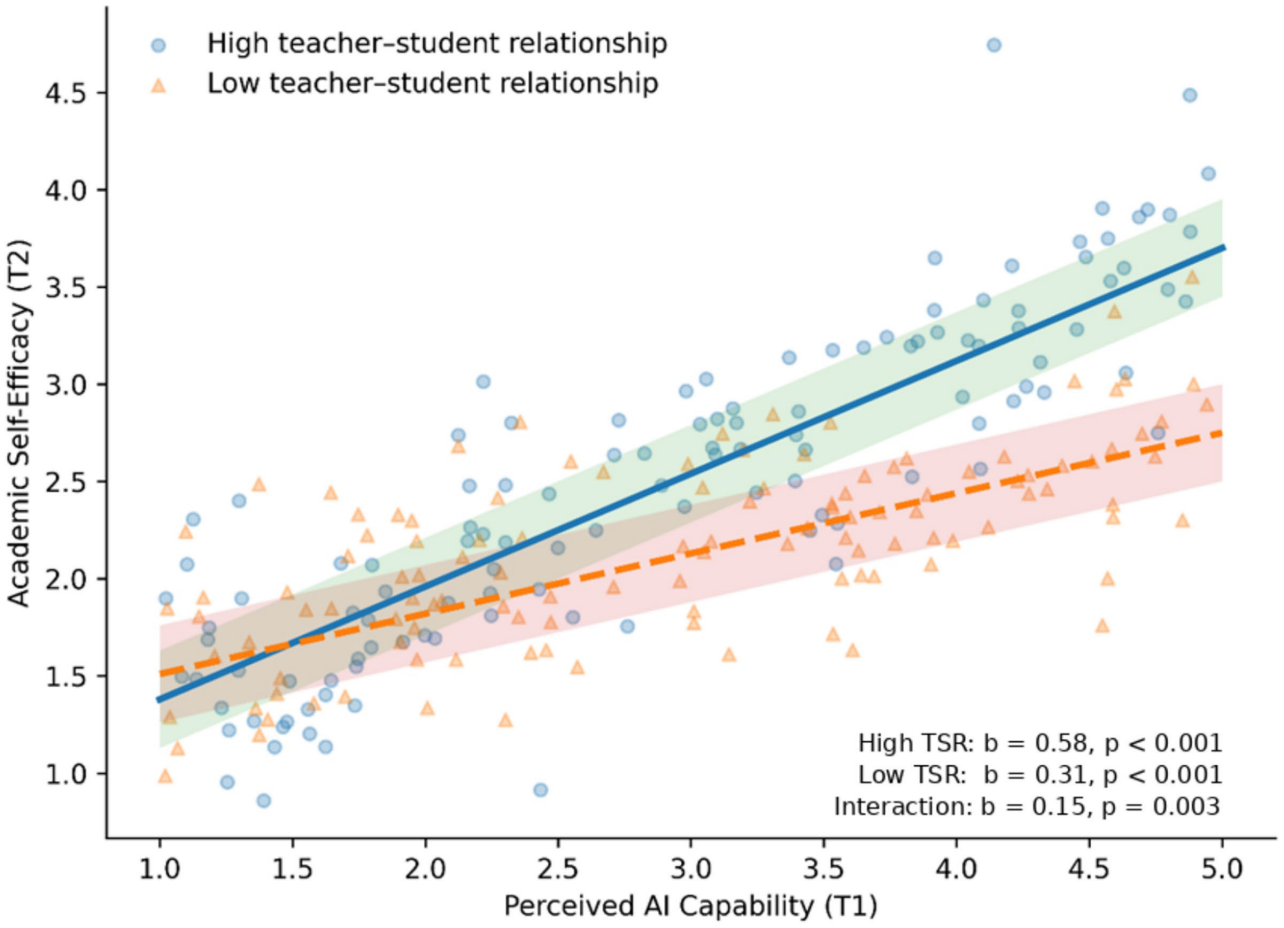
Conditional relationships between perceived AI capability and academic self-efficacy, moderated by teacher-student relationship. Conditional effects of perceived AI capability on academic self-efficacy at different levels of teacher-student relationship. The solid and dashed lines represent the regression relationships under conditions of high and low teacher-student relationship, respectively. The shaded areas denote the 95% confidence intervals. Statistical values annotated in the figure correspond to the simple slope and interaction term test results, illustrating the moderating role of teacher-student relationship in this pathway.

Moderated mediation was further tested using bias-corrected bootstrap. Conditional indirect effects of PAC on LE via ASE differed significantly across TSR levels (Table 6). Under high TSR, the indirect effect was stronger (b = 0.28, Boot SE = 0.05, 95% CI [0.18, 0.38]); under low TSR, it was weaker (b = 0.15, Boot SE = 0.04, 95% CI [0.07, 0.23]). The difference between conditions was significant (Δb = 0.13, 95% CI [0.04, 0.22]), confirming that TSR moderated the indirect path (Figure 5).

In summary, the results support the moderating role of TSR on the PAC–ASE link and establish a moderated mediation model, suggesting that positive TSR amplify the positive cascading effects generated by AI capability perceptions in smart education environments.

## 4 Discussion

This study focused on how learners’ subjective perceptions of AI capability are temporally associated with their subsequent learning engagement, further examining the mediating mechanism of ASE and the contextual boundary role of TSR. Employing a three-wave longitudinal design and structural equation modeling, the study temporally separated the measurement of key variables to provide rigorous mechanistic evidence for how technological capability cues in smart education are transformed into learners’ psychological resources and ultimately manifest as differences in learning engagement.

Overall, the sample exhibited moderately high levels of PAC and TSR, with cross-temporal correlations showing consistent directions. This pattern provides an empirical basis for subsequent structural path analysis and suggests that technological and social-relational variables jointly constitute learners’ resource ecology in smart education contexts.

This study found a stable positive association between PAC and learning engagement, with the direct effect remaining significant after including the mediator (*β* = 0.21), consistent with a partial mediation structure. This finding supports viewing PAC as an effective contextual cue that operates not only through psychological resource pathways but also via more direct routes, such as enhancing task completion efficiency and reducing cognitive load to promote sustained investment. Importantly, the significant direct and indirect effects of PAC on learning engagement, even after controlling for prior AI experience and academic achievement, suggest that perceived AI capability is not reducible to general technology acceptance or system quality perceptions. Instead, PAC functions as a capability cue that learners actively interpret and internalize, consistent with social cognitive theory’s emphasis on environmental signals shaping self-beliefs. This finding extends prior research that primarily focused on whether AI is accepted or useful, by explaining how perceived capability translates into sustained learning investment. Consistent with this, recent empirical research on AI-driven personalized feedback shows it can enhance LE by improving goal-related process variables and self-efficacy (Han, 2025; Huang and Wang, 2023; Xu et al., 2025), suggesting a relatively direct link between technological capability cues and learning investment (Wang et al., 2025). The moderate size of the direct effect is theoretically meaningful, implying that merely enhancing perceptions of system capability may be insufficient to significantly alter LE over longer timescales. Compared to models using technology adoption as outcomes, learning engagement—as an outcome more closely tied to learning process quality—is typically more constrained by individual beliefs and social context, giving technological cues a natural ceiling. In terms of practical significance, a standardized coefficient of *β* = 0.21 is modest by conventional benchmarks in educational psychology (Cohen, 2013), but it is comparable to effect sizes reported in recent meta-analyses and empirical studies on technology-enhanced learning and engagement. For instance, a meta-analysis (Heung and Chiu, 2025) on ChatGPT and student engagement found that ChatGPT-based learning produced medium to large effect sizes across behavioral, cognitive, and emotional engagement dimensions compared to non-ChatGPT learning. Similarly, research on AI-driven personalized feedback (Xu et al., 2025) has demonstrated significant positive relationships with goal achievement, academic self-efficacy, and learning engagement. Thus, the observed effect is practically meaningful in the context of AI-supported learning, where multiple factors (e.g., instructional quality, peer influence, prior achievement) concurrently shape engagement. A *β* of 0.21 implies that a one-standard-deviation increase in perceived AI capability is associated with a 0.21-standard-deviation increase in learning engagement, which, over a semester, could translate into noticeable differences in students’ sustained investment and academic behaviors.

What distinguishes the present model from prior self-efficacy mediation studies is the specific nature of the antecedent—PAC—and its interaction with the social-relational context in AI-augmented learning. Unlike conventional predictors such as perceived teacher support or task clarity, PAC reflects learners’ judgments about an algorithmic agent’s competence. This judgment entails novel cognitive appraisals, such as assessing whether the AI’s suggestions are trustworthy, whether its adaptations respect learner autonomy, and whether its capabilities complement or substitute for the learner’s own efforts. The finding that TSR moderates the PAC→ASE path further underscores the novelty: the same AI capability cue produces stronger self-efficacy gains when students perceive high-quality teacher-student relationships. In other words, AI does not function in isolation; its psychological effects are systematically shaped and amplified by human relational resources. This socio-technical interaction mechanism has not been documented in traditional self-efficacy models, which typically treat environmental cues as either human-only or static. Therefore, the study contributes not just a contextual application but a theoretically grounded extension into human-AI collaboration.

This study found support for a mediating role of ASE between PAC and learning engagement, with mediation accounting for nearly half of the total effect, highlighting the centrality of psychological resources in smart education processes. Results showed significant effects of PAC on ASE (*β* = 0.42) and ASE on LE (*β* = 0.45). This evidence aligns with recent conclusions positioning self-efficacy as a hub in engagement mechanisms; studies in generative AI learning contexts have found links between enhanced critical thinking and self-efficacy, revealing the role of psychological process variables in shaping learning outcomes (Shahzad et al., 2025; Liang et al., 2023). Notably, unlike studies treating AI tools as external factors directly boosting performance, this study suggests a more interpretable pathway: capability cues first influence learners’ judgments of their own learning ability, which then drive the continuity and depth of learning activities. This explanation aligns with social cognitive theory and accounts for the heterogeneity observed in practice. The proportion mediated (48%) indicates that while self-efficacy is a key mechanism, a substantial portion remains unexplained, potentially involving self-regulation strategies, goal orientation, or judgments about AI output credibility and metacognitive monitoring (Sufyan Ghaleb and Alshiha, 2023). Recent research on student AI literacy indicates that such competencies may jointly determine engagement quality alongside self-efficacy (Bećirović et al., 2025). Thus, the partial mediation structure reflects a realistic portrait of smart education effects as multiple concurrent pathways, with self-efficacy as one of the most stable and intervenable hubs.

The most significant finding is the evidence for a moderating role of TSR on the PAC–ASE path and establishing moderated mediation evidence. The interaction term was significantly associated with ASE (*β* = 0.12). Simple slopes indicated that the positive association between PAC and ASE was stronger under high TSR conditions than under low TSR conditions. Conditional indirect effects also differed significantly, consistent with the idea that social relational resources may influence how technological capability cues relate to psychological resources. This finding is consistent with recent evidence on teacher support, self-efficacy, and learning outcomes; structural model research on generative AI-assisted learning has indicated that teachers’ supportive behaviors directly predict learners’ ASE and indirectly affect learning outcomes through self-efficacy (Fang et al., 2025). This study extends that logic to smart education: TSR not only directly benefits learners but also is associated with whether students can view AI system capabilities as usable resources and internalize them as a sense of competence and control. A positive TSR may reduce learners’ uncertainty and anxiety associated with AI feedback, making them more likely to perceive AI suggestions as actionable learning support rather than external control. Studies across educational contexts have found moderation or mediation mechanisms involving TSR and self-efficacy (Asare, 2025). Critically, while some AI education research emphasizes AI’s autonomy and immediate feedback as sufficient to boost motivation, the moderated mediation evidence here suggests that overlooking TSR may lead to overestimating the universal effect of technological capability itself and underestimating the constraints social context places on realizing technological value. For current university practices promoting AI tool governance, this aligns with survey findings of student uncertainty about AI usage norms (Zhao et al., 2025), indicating learners need explanatory support from teachers to reduce uncertainty and form stable strategies.

Regarding the multidimensional nature of learning engagement, modeling it as a higher-order latent variable comprised of behavioral, emotional, and cognitive dimensions helps mechanistically understand the implications of this study’s results. Technological capability cues may more directly influence behavioral engagement (e.g., promoting task completion and practice frequency), while exerting a stronger indirect push on cognitive engagement via self-efficacy (e.g., increasing deep processing and strategy use). Emotional engagement may rely more on relational support and positive emotional experiences during learning. Although this study did not separately estimate differential paths for the three dimensions in the structural model, theoretical consistency suggests a typically stronger association between self-efficacy and cognitive engagement, as self-efficacy directly affects persistence and strategic investment when facing complex tasks. Recent research on generative AI promoting feedback engagement emphasizes that whether AI as a feedback tool fosters active participation depends on whether learners engage in meaning-making and self-regulation processes concerning the feedback—a mechanism closer to cognitive engagement (Zhan et al., 2025). It is also important to avoid equating PAC simplistically with improved learning quality. Some studies have warned that excessive dependence on AI tools may be associated with reduced critical thinking and lower-quality cognitive engagement, even when students appear to participate more frequently or complete tasks more efficiently (Tian and Zhang, 2025). This points to the need for future research to incorporate qualitative aspects of engagement, more finely distinguishing high- from low-quality participation, and examining through which psychological processes AI capability cues affect different types of engagement. Nevertheless, the current study’s higher-order modeling approach does not test these differential pathways directly; dimension-specific analyses are needed in future research to validate these conjectures.

Control variable results provide supplementary insights for understanding the mechanisms. Gender showed weak and mostly non-significant associations with main variables in the sample. Grade level showed a weak positive correlation with learning engagement. Prior academic achievement showed significant but moderate-weak associations with both self-efficacy and learning engagement. These patterns align with the commonsense understanding that learning investment is related to, but not wholly determined by, existing ability foundations. They also support this study’s conceptualization of self-efficacy as a psychological resource shaped by contextual cues. Notably, prior experience with intelligent learning systems correlated 0.24 with PAC. This suggests that PAC is not purely determined by objective system performance; learners’ technical experience and usage scripts may influence their capability judgments, subsequently affecting psychological and behavioral outcomes. Structural model research in related fields similarly indicates that factors like technological readiness and self-efficacy influence AI tool use and attitudes (Saihi et al., 2024; Shi et al., 2025). Therefore, in practical terms, fostering positive student perceptions of system capability may require simultaneous attention to user training and usage context design, not merely relying on system feature upgrades.

The findings yield several actionable implications for practice. First, for intelligent system design, developers should incorporate transparent and explainable feedback mechanisms that help learners accurately calibrate their self-efficacy. Systems that merely provide correct answers may inadvertently foster over-reliance; instead, features such as process-oriented guidance or Socratic questioning can support learners’ competence development while preserving their sense of agency. Second, for instructors, the findings underscore the importance of proactively interpreting AI-generated suggestions and clarifying the appropriate scope of AI use. Teachers can serve as interpretive guides who help students transform AI capability cues into usable learning resources. Simple strategies—such as discussing AI output limitations in class, modeling effective prompting techniques, or providing timely encouragement—can significantly amplify the positive effects of AI tools on students’ self-efficacy. Third, for institutional policy, universities should not treat AI adoption as a purely technological initiative. Professional development programs for faculty should address not only technical skills but also strategies for maintaining supportive teacher-student rapport in technology-enhanced settings. When implementing AI systems across courses, institutions should consider pairing such rollouts with structured opportunities for teacher-student interaction, ensuring that technological integration does not inadvertently weaken the relational foundations of learning.

Regarding the magnitude of the observed effects, the direct path from PAC to LE and the indirect path via ASE are modest in absolute terms but educationally meaningful. Cohen (2013) guidelines for *β* in path analysis (0.10 = small, 0.30 = medium, 0.50 = large) suggest these effects are in the small-to-medium range. However, in field studies of learning engagement—where myriad uncontrollable factors (e.g., prior knowledge, motivation, classroom dynamics) operate—effects of this size are typical and can have cumulative practical impact when aggregated across students and time. For example, an effect of *β* = 0.21 means that students who perceive AI as highly capable are likely to score approximately 0.2 standard deviations higher on learning engagement than those with lower PAC. In practical terms, this could correspond to a difference of several percentage points in assignment completion rates, time-on-task, or self-reported deep learning strategies. Moreover, the indirect effect accounting for 48% of the total effect underscores the importance of self-efficacy as a leverage point: interventions designed to boost both PAC and ASE could yield larger combined effects than targeting either alone.

It is also important to consider the contextual dependency of the findings. The moderated mediation model revealed that teacher-student relationship significantly shapes the PAC→ASE link. This implies that the same level of perceived AI capability may produce different psychological and behavioral outcomes depending on the quality of social-relational resources available to learners. Furthermore, the single-university sample likely shares certain institutional characteristics (e.g., teaching norms, technology integration policies, student demographics) that may interact with AI capability perceptions. For instance, in institutions where AI use is highly regulated or where teacher-student interactions are more formal, the observed effect sizes might differ. Thus, the results are not claimed to be universal but rather illustrative of mechanisms that may operate differently across contexts. Future research should systematically vary contextual features (e.g., AI system transparency, institutional support, class size) to map the boundary conditions of the model.

In summary, PAC is not merely a relabeling of perceived usefulness, system quality, or AI trust. Its theoretical value lies in capturing the learner’s interpreted capability signal from AI, which directly feeds into self-efficacy formation—a mechanism largely absent in traditional technology acceptance models. This distinction is empirically supported by the discriminant validity results and theoretically reinforced by the moderated mediation model involving teacher-student relationship.

Several limitations should be acknowledged when interpreting the findings. First, the study’s findings should be interpreted with caution due to both methodological and contextual constraints. Methodologically, although a three-wave longitudinal design was employed, the significant reverse path from ASE to PAC suggests potential bidirectionality, precluding strong causal claims. The absence of cross-lagged panel modeling (CLPM) means that the results demonstrate temporal associations rather than definitive causal ordering. Contextually, the data were collected from a single university using the same AI system, which limits generalizability to other institutions, disciplines, AI platforms, and cultural settings. Future research should employ CLPM or experimental designs to clarify directionality, and replicate the model across diverse educational contexts to assess the robustness of the observed effects. Second, all core variables were assessed via self-report measures. Although the three-wave design temporally separated the measurement of independent, mediator, and dependent variables—and statistical tests (e.g., comparing the theoretical model with a single-factor model) indicated that common method bias was not a major threat—social desirability and recall biases may still influence responses. Future studies could complement self-reports with objective behavioral data, such as system log files or trace data capturing actual interaction patterns with AI tools, to provide a more comprehensive picture of learning engagement. Third, TSR was measured at Time 1 as a relatively stable contextual resource. However, this relationship may evolve dynamically over the course of a semester, particularly as teachers and students navigate new technologies together. The current design does not capture such dynamic changes. Future research employing more intensive longitudinal designs—such as weekly diaries or experience sampling methods—could model the co-evolution of relational resources and technological cues, offering deeper insight into how social context interacts with AI perceptions over time. Fourth, although learning engagement was modeled as a higher-order latent variable encompassing behavioral, emotional, and cognitive dimensions, the study did not systematically examine whether PAC differentially influences these three dimensions. Given that these dimensions are theoretically and empirically distinct, they may function differently in AI-supported learning contexts. For instance, PAC might have a stronger direct effect on behavioral engagement (e.g., time spent, task completion) by reducing friction and providing immediate feedback, whereas its effect on cognitive engagement (e.g., deep processing, strategy use) may be more strongly mediated by academic self-efficacy and may also be moderated by teacher-student relationship. Emotional engagement, in turn, could be more sensitive to social-relational factors than to AI capability cues. The current second-order approach treats engagement as unidimensional for the purpose of testing the overall mediation model, but this aggregation may mask dimension-specific pathways. Future research should disaggregate engagement dimensions and examine potential differential effects of PAC, ASE, and TSR on behavioral, emotional, and cognitive engagement separately, possibly using dimension-specific mediation or moderated mediation models.

Finally, the mechanism framework may be expanded. Variables such as AI literacy, metacognitive monitoring, and trust in AI outputs could influence both self-efficacy formation and the quality of learning engagement. Future studies could incorporate these constructs to build more comprehensive multiple-mediation or moderated-mediation models. Additionally, future research should examine whether PAC differentially affects behavioral, emotional, and cognitive engagement, as these dimensions may have distinct antecedents and consequences in AI-supported learning environments.

## 5 Conclusion

Grounded in social cognitive theory and conservation of resources theory, this three-wave longitudinal study found that PAC at Time 1 was positively associated with LE at Time 3 (*β* = 0.21, *p* < 0.001). ASE at Time 2 partially mediated this relationship (indirect *β* = 0.19, 95% CI [0.13, 0.25]), accounting for 48% of the total effect. Teacher-student relationship (TSR) at Time 1 significantly moderated the PAC–ASE link (β_interaction = 0.12, *p* = 0.003). The conditional indirect effect of PAC on LE via ASE was stronger under high TSR (b = 0.28, 95% CI [0.18, 0.38]) than under low TSR (b = 0.15, 95% CI [0.07, 0.23]), with a significant difference (Δb = 0.13, 95% CI [0.04, 0.22]). Practically, system designers should offer explainable, process-oriented feedback; instructors should clarify AI limitations and model effective use; institutions should pair AI adoption with teacher training to maintain supportive rapport. Limitations include a single-university sample, self-reported measures, and TSR measured only at T1. Overall, perceived AI capability temporally associates with learning engagement directly and indirectly via self-efficacy, and positive teacher-student relationships amplify this pathway, advancing smart education research toward mechanism-focused explanations that integrate psychological and relational conditions.

## Data Availability

The raw data supporting the conclusions of this article will be made available by the authors, without undue reservation.
